# Detection of distant evolutionary relationships between protein families using theory of sequence profile-profile comparison

**DOI:** 10.1186/1471-2105-11-89

**Published:** 2010-02-17

**Authors:** Mindaugas Margelevičius, Česlovas Venclovas

**Affiliations:** 1 Institute of Biotechnology, Graičiūno 8, LT-02241 Vilnius, Lithuania

## Abstract

**Background:**

Detection of common evolutionary origin (homology) is a primary means of inferring protein structure and function. At present, comparison of protein families represented as sequence profiles is arguably the most effective homology detection strategy. However, finding the best way to represent evolutionary information of a protein sequence family in the profile, to compare profiles and to estimate the biological significance of such comparisons, remains an active area of research.

**Results:**

Here, we present a new homology detection method based on sequence profile-profile comparison. The method has a number of new features including position-dependent gap penalties and a global score system. Position-dependent gap penalties provide a more biologically relevant way to represent and align protein families as sequence profiles. The global score system enables an analytical solution of the statistical parameters needed to estimate the statistical significance of profile-profile similarities. The new method, together with other state-of-the-art profile-based methods (HHsearch, COMPASS and PSI-BLAST), is benchmarked in all-against-all comparison of a challenging set of SCOP domains that share at most 20% sequence identity. For benchmarking, we use a reference ("gold standard") free model-based evaluation framework. Evaluation results show that at the level of protein domains our method compares favorably to all other tested methods. We also provide examples of the new method outperforming structure-based similarity detection and alignment. The implementation of the new method both as a standalone software package and as a web server is available at http://www.ibt.lt/bioinformatics/coma.

**Conclusion:**

Due to a number of developments, the new profile-profile comparison method shows an improved ability to match distantly related protein domains. Therefore, the method should be useful for annotation and homology modeling of uncharacterized proteins.

## Background

Common evolutionary origin or homology is one of the key concepts in biology. Homologous proteins usually share similar three-dimensional shape and often perform identical or similar molecular functions. Therefore, detection of homology is now routinely used to make inferences regarding structure, function or evolution for the protein of interest. Protein sequence comparison is the primary means for establishing homology. For closely related proteins, sequence similarity can be detected even by an untrained eye, however, the similarity becomes weak and difficult to distinguish from random as the evolutionary distance increases. In many cases not until three-dimensional structures become available the homology between proteins can be established.

Comparison of multiple sequence alignments instead of individual sequences can often facilitate inference of remote homology relationships. This should not be surprising, because in contrast to a single sequence, a set of aligned related sequences can tell much more about the conservation (functional or structural importance) of individual positions or regions within the polypeptide chain. For homology detection, multiple sequence alignments are generally converted into either of the two numerical forms: position-specific sequence profiles or Hidden Markov Models (HMMs) [1-7]. The main difference between the traditional sequence profile and HMM is that the latter incorporates position-specific insertion and deletion probabilities.

At present, profile-profile or HMM-HMM comparison represents arguably the most sensitive remote homology detection strategy. Although over the years profile-profile (HMM-HMM) comparison methods have improved significantly, they still lag behind methods based on protein structure comparison. On the other hand, there are a number of areas for potential improvement of such methods, including the way profiles (HMMs) are constructed, profile (HMM) similarity is scored and statistical significance of that score is estimated. For example, fixed gap opening and extension penalties, traditionally used in profile comparisons, is a poor representation of protein evolution. Thus, introduction of position-dependent variable gap penalties might be expected to lead to considerable improvement of profile-based methods. Further development of statistical framework is another promising area of improvement for both profile and HMM-based methods.

Here, we present COMA (**C**omparison **O**f **M**ultiple **A**lignments), a new profile-profile search and comparison method that has a number of novel features. However, the two features that distinguish COMA from other profile-profile comparison methods most, are position-dependent gap penalties and a global score system, which enables analytical solution of the statistical parameters used in estimation of the significance of a match. We show that at the protein domain level COMA performs better than several other state-of-the art homology detection methods.

## Methods

Methods that aim to detect relationship between protein families by comparing corresponding sequence profiles include three main components: 1) profile construction algorithm, 2) logic for profile comparison and 3) estimation of statistical significance of the profile-profile alignment score. In this section we describe underlying theoretical considerations and their implementation in COMA.

### Profile construction

Multiple sequence alignments are converted into profiles similarly as in PSI-BLAST [8], but with new additions and modifications as described below.

#### Filtering of high and low complexity regions

Sometimes multiple sequence alignments have stretches of positions (columns) with highly divergent residue distribution, which means that the expected occurrence for nearly all residue types is about the same. These stretches, defined here as high complexity regions, often indicate either misaligned or structurally unrelated sequence regions and may negatively affect subsequent comparison of profiles. Comparison problems may also be caused by multiple alignment regions of low compositional complexity. These may include groups of columns with short periodic repeats or very similar residue distributions and often correspond to unstructured regions of aligned sequences. To deal with both extremes of compositional complexity, the SEG algorithm [9] has been modified and adopted to work with multiple alignments and profile positional vectors. By default, COMA uses the modified SEG for filtering high complexity regions prior to the construction of a sequence profile. Optionally, low complexity regions can be filtered as well.

#### Sequence weighting

As in PSI-BLAST, sequence weights are computed from the reduced multiple alignment that is compiled for every position of the input multiple alignment. However, in contrast to PSI-BLAST, sequences from the multiple alignment are included into the reduced multiple alignment only if the residues contributing to the corresponding position are not at the sequence termini. This is important, because in some cases, the reduced multiple alignment may consist of only a single column, leading to the assignment of incorrect sequence weights and subsequently to a dramatic deterioration of the overall performance in profile comparisons. In COMA, more stringent requirements for reduced multiple alignments help to avoid such situations. After reduced multiple alignments are compiled, sequence weights are calculated by the modified Henikoff & Henikoff sequence weighting algorithm [10].

#### Initial scores and probabilities

Profile scores are calculated using an initial score table such as that from BLOSUM [11] or PAM [12] series. Here, we use a newly derived initial score table, constructed from multiple alignments obtained for ASTRAL [13] sequences. The table is derived using a method similar to that of Henikoff & Henikoff [11] and can be easily recalculated for a different set of multiple sequence alignments. Residue substitution frequencies and background probabilities derived from the initial score table are used to compute target probabilities (estimated frequencies of each residue at a given profile position) using the Tatusov pseudo count method [14].

#### Composition-based statistics

The alignment score obtained using explicitly defined score table is related to its statistical significance [15]. Thus, each score table used for generating alignments can be characterized by the statistical parameters of the score distribution [16]. In COMA, the computed statistical parameter values for the initial score table are used for composition-based statistics [17]. Once a profile is constructed, it is compositionally adjusted to have statistical parameter values equal to those of the initial score table. Such a compositional adjustment is needed to normalize profile scores so that alignment scores would follow the reference distribution.

#### Deletions and insertions

Comparison of profiles in many cases is bound to produce gapped alignments. Therefore, an accurate estimation of insertion and deletion probabilities at every profile position is a significant factor that affects alignment quality. Currently, profile-based methods such as PSI-BLAST [8] and COMPASS [5] use constant gap penalties, but that is a poor approximation of evolution of protein families. In COMA, insertion and deletion probabilities are allowed to vary along the length of the sequence profile. Deletion is defined as gap(s) in a sequence aligned against the residue(s) in another sequence, while insertion corresponds to residue(s) aligned against the gap(s). The probability of a deletion at the position *i *of the multiple alignment is expressed as 1-Σ_*a*_*f*_*a*_, where *f*_*a *_is the weighted observed frequency, calculated for the residue *a *at the position *i*. An exhaustive analysis of 4611 profiles showed that deletion probabilities can be accurately approximated linearly. Thus the generalized probability of a deletion at the profile position *i *is described by the linear function whose slope and intercept are determined from the probability values at the boundary positions of the deletion. The probability of an insertion at the profile position *i *is defined as the weighted observed frequency for the gap (*f*_-_) at the profile position *i*. Since the insertion always corresponds to a residue, which occupies a profile position, no generalization of probabilities of insertions is needed.

### Profile-profile comparison

In COMA, a pair of profiles is aligned using a modified Smith-Waterman dynamic programming algorithm [18], the score system and the position-dependent variable gap cost scheme.

#### Score system

The score *s*_*ia *_of the residue *a *at the profile position *i *is defined as , where  is the target probability of the residue *a *at the profile position *i, p*_*a *_is the background probability of the residue *a, λ*_*p *_is the reference statistical parameter of ungapped alignments of protein sequences such that the target probabilities  sum to 1 [8,16].

Similar log-odds principle is used to construct a score system for a pair of profiles. An individual profile is the score table constructed for a single sequence using the processed information from the multiple alignment. The information from the multiple alignment is in part expressed with the weighted observed frequencies  for the residue of type *a *at profile positions *i*. Weighted observed frequencies are important measures of positional conservation and are also used to derive the target probabilities . Thus, we use both profile quantities ( and ) to define the target probabilities when comparing a pair of profiles. Let  denote the target probability of the residue *a *at the profile position *i *of the first profile, and  the corresponding target probability at the profile position *j *of the second profile. Let  and  be the observed frequencies of the residue *a *at the profile positions *i *and *j *of the first and second profile, respectively. We say that two positions *i *and *j *of two distinct profiles are more similar if the probability odds ratio  is greater. The score *s*_*ij *_of matching the position *i *of the first profile with the position *j *of the second profile is calculated as follows:(1)

where *N *is the normalizing term such that the background probabilities for a pair of profiles sum to 1:(2)

*λ *in Equation 1 is a statistical parameter of profile-to-profile ungapped alignments such that the target probabilities of a pair of profiles (numerator of the odds in Equation 1) sum to 1:(3)

where *p*(*s*_*ij*_) is the background probability for a pair of profiles or, in other words, probability of the score *s*_*ij*_, *s*_*k *_are the discrete values of *s*_*ij *_and(4)

Having all terms defined, we can express the score *s*_*ij *_from Equation 1 as follows:(5)

Note that the statistical parameter *λ *is solved from the Equation 3 using the background probabilities in Equation 4 so that the target probabilities for a pair of profiles in Equation 1 sum to 1. To solve Equation 3, scores *s*_*ij *_are multiplied by 2^*c *^(to rescue a precision of *c *bits) and rounded to the nearest integer value.

#### Correction of scores

Multiple sequence alignments, from which profiles are constructed, may carry very different amounts of evolutionary information. For example, the information extracted in one case from three and in other case from three hundred aligned diverse sequences cannot be considered to have the same value. Thus before the final alignment of a pair of profiles, assuming that probabilities do not change, scores *s*_*ij *_are corrected as follows:(6)

where *t*^(*i*) ^and *t'*^(*j*) ^are the effective number of sequences comprising a reduced multiple alignment at the profile position *i *of the first profile and position *j *of the second profile, respectively. Since the limit of the sum  is 2, the corresponding constant is introduced in the numerator of Equation 6 to keep both the corrected and original scores in a similar range. The parameter *λ *in Equation 6 is now solved from Equation 3 so that the corrected target probabilities of a pair of profiles sum to 1. Using the correction scheme (Equation 6), the profile scores obtained from a "thin" alignment are down-weighted in comparison to the scores from a "thick" alignment. In the general case the scores of the "thin" profile are averaged over the observed weighted frequencies of the "thick" profile using higher weights, while the scores of the "thick" profile are averaged over the corresponding frequencies of the "thin" profile using lower weights. If one of the two profiles, say the first, is constructed from a single sequence,  and then the log-odds of the first profile are averaged over the observed weighted frequencies of the second profile. Several other score correction schemes have been tested, but the one in Equation 6 worked best. In the extreme case, when both profiles are constructed from a single sequence, no correction is applied.

In addition to "thickness", the information content of the profile position *i *is characterized by the expression of relative entropy . Small values of *r*^(*i*) ^indicate uninformative and variable positions. Therefore, scores of the positions, having *r*^(*i*) ^lower than the specified threshold, are scaled down by the adjustable factor (0.5 by default). The threshold is inversely proportional to -log *E*, where *E *is the pre-calculated *E*-value per hit. For compositionally similar profiles this threshold will be low. Scaling down of the scores for positions of low entropy is performed independently of the effective number of sequences ("thickness") comprising corresponding positions.

#### Gap cost computation

Position-dependent gap costs for a pair of profiles are defined by the insertion/deletion probabilities for every position of individual profiles and by the limits within which position-specific gap costs are allowed to vary. The high scoring positions of the score system are mostly conserved, and the probability for a gap to occur there is low. In contrast, the positions of low scores often match variable regions (e.g. loops in protein structure) where the probability of a gap becomes higher. Thus, gap cost limits should be more stringent for high scoring positions than for low scoring ones. Following this logic, gap cost limits for the first and the second profile are calculated using respectively the row and column maximum scores of the score system. For each position of the score system, the number *w *of maximum scores is recorded and their autocorrelation is calculated. Since the autocorrelation is a sum of the products, the characteristic value of maximum scores for the corresponding position is calculated as the square root of the autocorrelation value divided by the number of summed products. This ensures that the characteristic value is within the similar range as the maximum scores. The final gap cost limit for the corresponding position is computed as the square root of the autocorrelation function of the characteristic values divided by the number of summed products. The goal of this procedure is to make the gap cost limit for the corresponding position dependent on the adjacent characteristic values. The formal description of this logic follows below.

Let *s*_*ij *_denote the score for the positions *i *and *j *of the score system. Let  be the sorted periodic sequence of the *w *maximum scores at the position *i *of the score system such that , , , .... Similarly, let  be the sorted sequence of the *w *maximum scores at the position *j *such that , , , .... Let further {*a*_*i*_}_*i *_be the periodic sequence such that  where  is the autocorrelation function, and let  be the periodic sequence defined so that  for all *i *(window size *ω *> 0). Similarly, let  be the sequence such that , where  and  be the periodic sequence defined for all *j*: . Then the gap cost limit *A*^(*i*) ^for the first profile computed at the position *i *of the score system is defined as follows:(8)

where  is the autocorrelation function of  in a window of size *k *(by default, *w *= *ω *= 4); *ω *(*ω *+1)/2 is the number of the summed products. If the condition in Equation 8 is not satisfied, *A*^(*i*) ^is assigned to 0.

Similarly, we define the gap cost limit *B*^(*j*) ^for the second profile at the position *j*:(9)

where  is the autocorrelation function of  in a window of size *k*. If the condition in Equation 9 is not satisfied, *B*^(*j*) ^is assigned to 0.

Sometimes low positive scores in the score system imply relatively low gap cost limits leading to high-scoring local alignments, which may be poor because of the positive expected score per aligned pair of positions [19]. In COMA, to address this problem, the term *z *is added to the multipliers within the autocorrelation functions in Equations 8 and 9 so that the gap cost limits are widened:(10)

The parameter *z *in Equation 10 is introduced twice in a two-step iterative protocol. In the first step, the term *z*, inversely proportional to √*H*, is added to each of the autocorrelated values. *H *is a statistical parameter of entropy (see statistical significance below) and its low values indicate low scores. By default, *H *is derived analytically for a pair of profiles (optionally, *H *may be computed for every individual position of the score system, thus increasing specificity at the expense of sensitivity). In the second step, an initial alignment and its *E*-value are computed. Based on this initial alignment, *z *is reevaluated as *-y*/(log *E *+ *x*), where *x *and *y *are adjustable parameters and *E *is the initial estimated *E*-value per hit. This reevaluation implies narrowing of gap cost limits in case of small initial *E*-values that indicate possibly related profile pairs.

A gap cost at a certain position of the score system is two-sided. It depends on both the deletion probability in the first profile and the insertion probability in the second (the deletion probability expresses probability for a gap to occur in the same profile, while the insertion probability - in the second). The final gap cost *G*^(*i*) ^for the first profile at the position *i *of the score system is defined as a fraction of the size of the gap cost limit *A*^(*i*) ^at the position by the superposition of the deletion and insertion probabilities:(11)

where *D*^(*i*) ^is the deletion probability of the first profile at the position *i, I*^(*j*) ^is the insertion probability of the second profile at the position *j*. Since the gap cost limits *A*^(*i*) ^by definition mean the costs of the deletions for the first profile, a weight of the deletion probability *D*^(*i*) ^of that profile in the expression for *G*^(*i*) ^is reduced by the constant *c *(by default *c *= 0.6). The gap cost for the second profile *C*^(*j*) ^is computed in a similar way:(12)

where *P*^(*j*) ^is the deletion probability of the second profile at the position *j, J*^(*i*) ^is the insertion probability of the first profile at the position *i*.

If multiple sequence alignment is "thin", the effect of deletion and insertion probabilities may be disproportionally large. For example, consider a position of multiple sequence alignment consisting of just two aligned sequences where the second sequence contains a gap. The insertion probability for that position would be 50%. To avoid such situations, the insertion/deletion probabilities are adjusted by a factor of 1/(1 + exp(-*t u *+ *v*)), where *t *is the effective number of sequences in multiple sequence alignment at a given position, and *u *and *v *are the adjustable parameters. When the alignment is "thick" (the effective number of sequences is large), the factor approaches 1 and the adjustment essentially has no effect.

All the heuristic parameters (*x*, *y*, *u*, *v*, *c,...*) were optimized on the most structurally diverse and most difficult subset (200-300 profiles) of the dataset.

### Statistical significance

An accurate estimation of statistical significance of the alignment score is important as it tells whether aligned sequence profiles (representing protein families) are likely to be related or not.

We use analytical estimation of statistical significance. The analytical approach was initially proposed [16] for any score table used for ungapped sequence alignments and meeting two necessary conditions: the mean of scores must be negative and at least one positive score must exist in the table. It was shown [20] that the distribution of the optimal alignment scores using a score table that meets these two conditions can be well approximated by the extreme value distribution (EVD). Furthermore, it was shown [19] that the limit of average score per aligned pair of positions exists for gapped alignments. This means that the theory can be extended for gapped alignments and this has been demonstrated in practice [8].

#### Statistical parameters

If the distribution of the optimal alignment scores is approximated by EVD, the expected value *E *(the expected number of alignment scores greater than or equal to *s *per database search) can be expressed as follows:(13)

where *m *is a length of the query profile, *n *is a length of the database. This expression of *E*-value and the corresponding *P*-value (*P *= 1-exp(-*E*)) are the ones used in COMA to estimate statistical significance. Since the score system used in COMA is amenable to the Karlin-Altschul statistics, the statistical parameters are derived as follows: the scale parameter of the distribution *λ*, is solved from Equation 3, where the scores *s*_*k *_are calculated from Equation 6, *K *is found by the formula given in [16]. Using the definitions of the target and background probabilities in Equation 1, the parameter of relative entropy *H *required to compute *K *is expressed as follows:(14)

It follows from Equation 1 that the target probabilities are equal to  where , and the log-odds in Equation 14 are equal to *λ s*_*ij*_. Using the notations found in Equations 3 and 4, *H *is expressed as:(15)

It is important to note that the search space *m n *used in Equation 13 is valid asymptotically. Thus, the edge effect (dependence of the expected alignment length on the alignment score) is corrected by computing additional statistical parameters *α *and *β *[21].

#### Composition-based statistics and the global score system

In COMA, statistical parameters are computed for each pair of profiles to be aligned. The exact values of solved statistical parameters (*λ, H, K*) may differ from pair to pair of profiles leading to adverse effects such as different statistical significance values for identical scores. To deal with this problem we apply composition based statistics using a newly introduced global score system. The idea of the composition-based statistics [17] is to transform a score table (the score system in the case of profiles) so that its statistical parameter *λ*_u_^*^ becomes equal to the reference value of *λ*_*u*_, where *λ*_*u *_is the statistical parameter of the distribution of ungapped alignment scores. For sequences the reference parameter corresponds to the known parameter of the residue substitution score table used, e.g. one of the BLOSUM [11] or PAM [12] families. For profiles, there is no explicitly defined alphabet of finite size such as that for sequences (twenty different residues).

**Figure 1:**
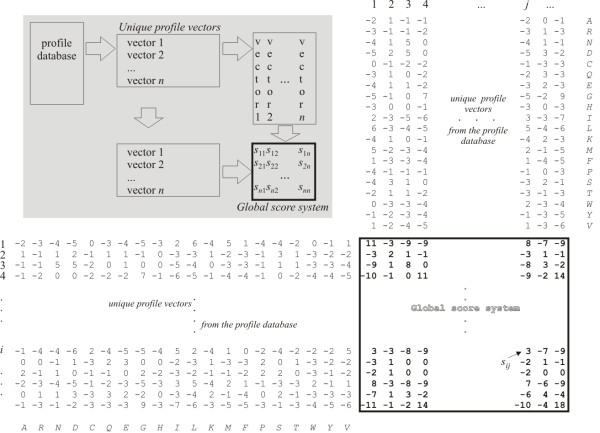
**The global score system of profile vectors**. Each cell within the global score system corresponds to the score *s*_*ij *_calculated using information from two profile vectors found at the row and column positions *i *an *j*, respectively.

Here, to make such an alphabet for profiles, we compile all unique profile vectors from the profile database used for profile comparisons and construct a global score system of profile vectors (Figure 1). Each cell (*i*, *j*) of the global score system contains the similarity score *s*_*ij *_of two profile vectors. Solving Equation 3 for the parameter *λ *gives us the value of the required reference parameter *λ*_*u*_. After the score system is constructed for a pair of profiles, its scores are transformed so that the parameter *λ*_u_^*^ of the score system becomes equal to the reference parameter *λ*_*u*_. Making use of the global score system involved a non-trivial algorithmic approach (to make it feasible, we introduced a two-level hash system with the binary search structures), but it significantly improved the performance of COMA.

#### Statistical significance of gapped alignments

The analytical theory of statistical significance estimation has been developed only for ungapped alignments [16]. Since there is no universal theory for gapped alignments, the statistical parameters of the score distribution of gapped alignments have to be estimated empirically. Although in theory [22] it is possible to derive analytical expressions of the statistical parameters of gapped alignments, in practice this does not seem to be feasible and, to our knowledge, so far has not been implemented. The gap cost scheme in COMA is complex, and the analytical approach in this case would not be feasible as well.

On the other hand, it has been shown empirically that the scores of gapped alignments are also distributed according to EVD [8]. For profile-profile comparisons, the score distribution might be slightly diverged from EVD [23] due to specifics of the profile scoring and gap introduction methods. However, for significance estimation the most important is the tail of the distribution, which can be accurately represented by EVD.

To derive the reference statistical parameters *λ*_*g *_and *K*_*g *_for gapped alignments, we compiled a set of unrelated COMA hits. This set comprises 160 513 hits that fulfil all of the following conditions: (a) profiles are from different SCOP [24] superfamilies, (b) COMA alignment score is ≥ 60, and (c) TM-score [25] according to the DALI [26] alignment evaluated in the local mode is ≤ 0.17 (see "Results" for detailed description of alignment evaluation).

**Figure 2:**
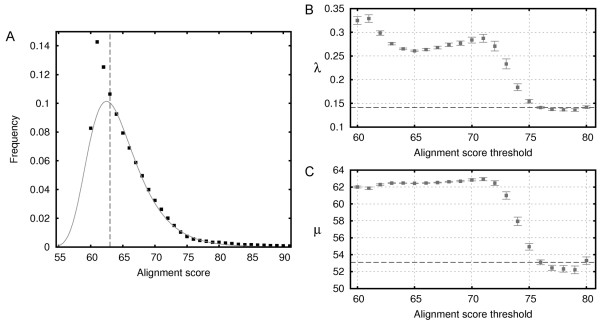
**Estimation of statistical parameters for score distribution of gapped alignments**. (A) Obtained alignment score distribution of unrelated hits; COMA scores are fitted to the extreme value distribution considering only the hits with the score of at least 63 (the dashed line). (B) Estimation of the scale statistical parameter *λ *and (C) the localization statistical parameter *μ *. Standard errors of the estimates are shown with error bars. The dashed line denotes the level at which the fluctuation of the statistical parameters becomes small.

Having compiled the set of unrelated hits, we then derive the scale and localization reference statistical parameters of the extreme value distribution, *λ *and *μ*, respectively, as follows. We progressively increase the lower bound of the alignment score threshold for alignments to be considered, each time re-estimating the statistical parameters for the resulting set. In this way we obtain a series of values for both *λ *and *μ *that in general show a significant variation (Figure 2). However, with the increase of alignment scores, the statistical parameter values become fairly stable (for scores over 75), allowing to select them as the reference values for gapped alignments (*λ *serves as *λ*_*g*_, while *μ *is used to calculate *K*_*g*_). In other words, the empirically derived reference values of *λ *and *μ *are expected to accurately describe the statistics of score distribution in the region of higher scores (corresponding to the tail of EVD).

As a result of the composition-based statistics, the parameter *λ *_u_^*^ solved and scaled for a pair of profiles is equal to the reference parameter *λ*_*u *_of the global score system: *λ *_u_^*^ ≡ *λ*_*u*_. Only in rare cases, e.g. because of low absolute values of scores (thus indicating weak signal of similarity if any), *λ*_u_^*^ and *λ*_*u *_might be slightly different. We then assume that the statistical parameter *λ*_u_^*^ of the score system for the pair of the profiles to be aligned with gaps differs from the reference parameter *λ*_g_^*^ exactly in the same ratio as *λ*_u_^*^ differs from *λ*_*u*_. The same principle is applied for estimation of the statistical parameter *K*_g_^*^ of the score system for the pair of profiles to be aligned with gaps. However, in contrast to *λ*, the values of *K*_*u *_and *K*_*u *_are of ten different. Thus, K_g_^*^ is calculated by adjusting the empirically estimated *K*_*g *_by the factor *K*_u_^*^/*K*_*u*_. The final expressions of *λ*_g_^*^ and *K*_g_^*^ are as follows:(16)

## Results

To evaluate COMA, we compared its ability to detect remote relationships and produce accurate alignments with that of other state-of-the-art profile-based methods. We chose COMPASS [5] (version 2.42), another profile-profile comparison method, HHsearch [6] (1.5), a method based on HMM-HMM comparison, and PSI-BLAST [8] (2.2.15), a widely used profile-sequence method.

### Test set

The assessment of distant relationships and corresponding alignments can be best done having three-dimensional (3D) structures of the test set proteins in hand. Here, we used SCOP [24] (1.71) protein structures and corresponding ASTRAL [13] sequences. Having focused on distant homology we only considered sequences 20% or less identical to each other. We also excluded sequences of small and membrane protein classes ('g' and 'f' respectively). As a result, we ended up with a large statistical sample consisting of 4611 sequences representing a wide variety of protein structures and a challenging set for similarity detection. For each sequence from this set, multiple alignments were built by running up to 6 iterations of PSI-BLAST against the NCBI non-redundant (nr) sequence database filtered to the maximum identity of 70%. To prevent unrelated sequences from entering the alignments, we ran PSI-BLAST using a stringent (10^5^) sequence inclusion threshold and with low complexity filter turned on. The resulting alignments were used in all-to-all comparison for all methods except for COMPASS. Before running COMPASS, alignment columns corresponding to gaps in the first (query) sequence were removed, substantially improving its performance.

### Evaluation framework

Traditionally, homology detection methods have been evaluated against a reference protein classification scheme and reference alignments serving as "gold standard". However, the more remote relationships are considered, the less straightforward becomes the use of "gold standard" classification schemes and alignments. For example, the original hierarchical SCOP classification scheme implies that protein domains assigned to different folds are unrelated and therefore should be considered as a false positive match in the benchmark. Yet, by now, it is commonly accepted that there are many evolutionary related proteins classified as having different folds. Perhaps most obvious examples include Rossmann-like domains, immunoglobulin-like β-sandwiches and β-propellers. Evolutionary relationships can be found even at the highest hierarchy level, between domains belonging to different SCOP classes (e.g. [27]).

Reference alignments usually have to be derived separately, using some structure comparison method, e.g. DALI [26]. However, different structure comparison methods tend to produce structure-based alignments that differ in coverage and/or accuracy. This problem is essentially non-existent for closely related proteins, but becomes very important in the cases of remote homology, the area specifically targeted by profile-profile comparison methods.

Therefore, to bypass additional problems with reference classification and reference alignments we chose to use exclusively the reference-free evaluation framework based on structural models. In this framework, no other information except for the pairwise sequence alignment produced by the assessed method and 3D structures of aligned protein domains is needed. Alignment is used to generate a 3D model of the query sequence based on the structure of the match. The questions of whether the match is correct (are aligned protein structures similar?) and whether the alignment is accurate are automatically answered by evaluating the obtained model against the real structure. Such model evaluation framework has been very effective in Critical Assessment of Techniques for Protein Structure Prediction (CASP) experiments lasting over a decade [28]. For assessment of homology detection methods the reference-free evaluation initially was introduced as a way to take care of exceptions within the "gold standard" SCOP classification [6] and, more recently, validated as an entirely self-sufficient evaluation framework [29].

Here, pairwise alignments obtained by every tested method are converted into corresponding 3D models using a standard MODELLER [30] (version 9.2) run. To evaluate the quality of the models we use the template modeling score (TM-score) [25], which is similar to GDT_TS [31] and MaxSub [32] measures, but is designed to be less dependent on the protein size. TM-score generates a single value in the [0; 1] range indicating how close is the model to the reference structure [25] (TM-score ≤ 0.17 implies a random match; TM-score ≥ 0.4 indicates a statistically significant similarity; for identical structures TM-score = 1). Accordingly, in our evaluation scheme the aligned pair of proteins is accepted as a true positive match, if the TM-score is ≥ 0.4 and a false positive match, if the TM-score ≤ 0.17. Alignments producing intermediate TM-score values (0.17 < TM-score < 0.4) are considered to represent "unknown" relationships.

### Evaluation of methods in all-to-all comparison

To measure the performance of all the evaluated methods, we performed all-to-all comparison for the test set (4611 multiple alignments). Although in our evaluation framework the alignment, produced by a method being tested, by itself defines both the quality of the match and the alignment accuracy, two different evaluation modes, global and local, are possible. In a global mode, the alignment is evaluated with respect to the entire protein domain. This is equivalent to asking how useful the alignment is for generating a structural model of that domain. In a local mode, the alignment is evaluated within its boundaries. This mode measures how well a particular method can detect and align possibly short, but structurally similar fragments, without the requirement that these fragments come from overall similar domains. A caveat of this mode is that very short alignments would often score very high, independently whether the aligned fragments are related or not. Therefore, in the local evaluation mode we only consider fragments that include at least 15 aligned residues, the length that approximates the transition from individual secondary structure elements into supersecondary structure motifs.

All-to-all comparison generates two matches between proteins A and B (AB and BA), scores of which in general may slightly differ. To reduce the redundancy, for each evaluated method we only consider one of the two matches producing a better statistical significance value. In both evaluation modes for every aligned pair AB we consider two models, A' and B' (A' is a model of A built using the structure of B as the template, and B' is a model of B built using the structure of A). Although the same AB alignment is used to generate both A' and B', their corresponding TM-scores in general differ, because they correspond to different structure pairs AA' and BB'. Of the two TM-scores for the AB pair we take the better one.

**Figure 3:**
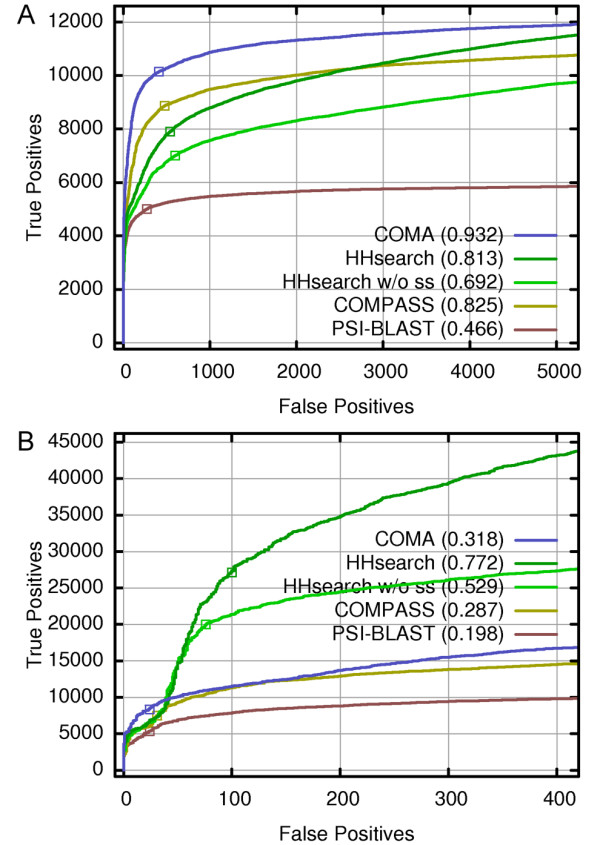
**Performance of the evaluated methods according to the ROC curves**. A match is defined as a true positive if a corresponding structural model gets TM-score ≥ 0.4, and a false positive if TM-score ≤ 0.17. HHsearch was tested both with and without (w/o ss) consideration of secondary structure information. Numbers in parenthesis indicate relative area under the corresponding ROC curve. (A) Global mode analysis, in which alignments are evaluated in respect to the entire structural domains. Empty squares mark approximate transition points, where false positives start accumulating more rapidly. Corresponding *E*-values for COMA, COMPASS, PSI-BLAST and HHsearch w/o ss are 0.01, 0.03, 0.0007 and 3e-6 respectively. HHsearch probability is 97.6% (B) Local mode analysis; evaluation is done within the boundaries of aligned segments; *E*-values at transition points for COMA, COMPASS, PSI-BLAST and HHsearch w/o ss are 8e-6, 1e-4, 1e-5, and 0.65 respectively. HHsearch probability is 65.3%. Correlation between the estimated and observed statistical significance values for each method from the purely statistical perspective without differentiating global and local modes is shown in Additional file 1, Figure S3.

Figure 3 shows the results of both global (Figure 3A) and local (Figure 3B) evaluation modes as Receiver Operating Characteristic (ROC) charts. In a ROC chart the number of true positives (TP) is plotted against the number of false positives (FP) after matches are sorted according to their statistical significance (e.g. by ascending E-values) for each method. The higher is the curve (or the larger is the relative area under the curve), the better is the performance of the method.

In the global evaluation mode, COMA is clearly better than the other methods included in the comparison. Within the high specificity range (low error rate) COMPASS is second, but in the lower specificity region (~26% error) is overtaken by the HHsearch version that uses secondary structure information. For the user of a particular homology detection method, the most important is the left-most region of the curve, because it corresponds to the statistically most significant matches. The significantly better performance by COMA in this region becomes even more apparent when the same data is plotted in a log-scale (Additional file 1, Figure S1A). To make sure that this result is not specific to the exact cutoff (TM-score ≥ 0.4) that we used as a criterion of true positive we have also tried alternative definitions (TM-score ≥ 0.35 and TM-score ≥ 0.45). In both cases we obtained qualitatively similar results with COMA being a top performer (Additional file 1, Figure S2A, C). In addition, we sought an independent means to confirm that the TM-score criterion is indeed sufficient to make a case for meaningful similarity of an aligned pair. For this, we analyzed the non-redundant set of true positives (according to the TM-score criterion) produced by all the evaluated methods. All pairs from the same SCOP superfamily as well as those coming from different superfamilies, folds or even classes, but producing DALI Z-score ≥ 2, were considered related. It turned out that in the global evaluation mode TM-score results are nearly perfectly reproduced by the combined SCOP/DALI classification scheme (Additional file 1, Table S1). In contrast, if only SCOP hierarchy is used for classification, the discrepancy is much larger.

The local evaluation mode measures the quality of the structural match predicted by the sequence alignment with respect to the aligned region only. The results of the local evaluation mode (Figure 3B; Additional file 1, Figure S2B, D) of HHsearch are strikingly different from those obtained in the global mode. Both versions of HHsearch, in particular the one using secondary structure information, outperform other methods by a large margin, except for the region of very high specificity, where COMA detects the largest number of true positives (Figure 3B; Additional file 1, Figures S1B, S2B, D). At the same time COMA, COMPASS and PSI-BLAST all show relative performance qualitatively similar to that of the global mode. Why then the HHsearch behavior is so much different in the local evaluation mode?

**Figure 4:**
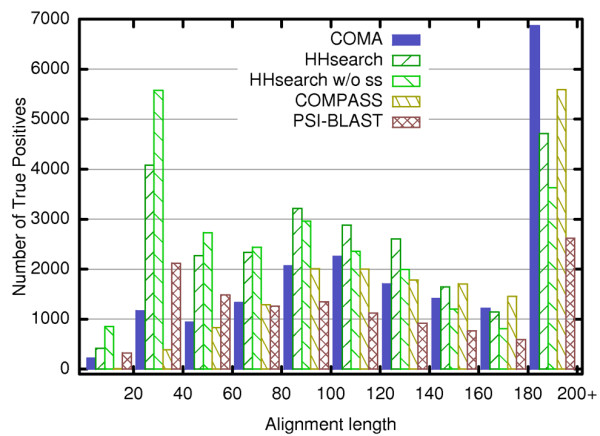
**Distribution of true positives according to the alignment length**. The figure shows distribution of true positives according to the length of corresponding alignments from the 30000 most significant hits found by each method. Only alignments including at least 15 aligned residues have been considered. Alignments over 180 in length are pooled together in the last bin.

The apparent reason for that becomes clear if we analyze the binned distribution of the length of alignments that score as true positives in the local evaluation mode for all the methods (Figure 4). Both versions of HHsearch strongly favor relatively short alignments (up to 40 aligned positions). In contrast, COMA and COMPASS, are both aimed at aligning longer segments. COMPASS specifically avoids short alignments (length <40). On the other hand, while COMA produces a significant number of short accurate alignments, its strength lies in generating long ones (over 180 aligned positions).

Thus the combined analysis of the performance by compared methods reveals the opposite preferences towards the alignment length by HHsearch on one side and COMA and COMPASS on the other side. It is interesting to note that for all of the compared methods the number of true positives is higher in local as opposed to the global mode analysis (Figure 3; Additional file 1, Table S1 and Figure S4). The SCOP/DALI criterion recaptures most of the true positives as defined by TM-score in the local evaluation mode, yet the disagreement is larger than in the global mode (Additional file 1, Table S1). This should not be surprising, because both SCOP and DALI are aiming at the global similarity between protein domains. Taken together the data indicates that 1) it is relatively easier to accurately align similar fragments than entire domains and that 2) a number of detected similar fragments are imbedded within domains that are not globally similar.

### Structure-based vs. profile-profile alignments

**Figure 5:**
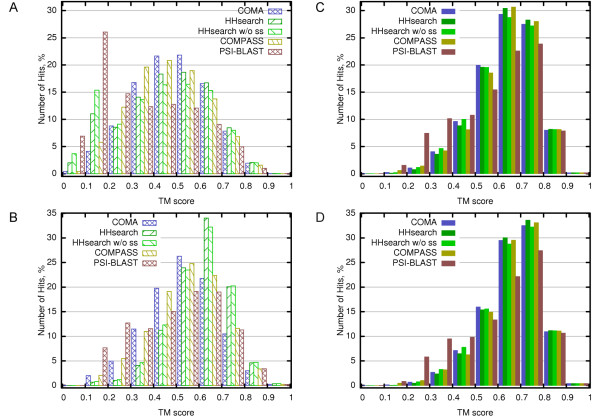
**Distribution of TM-scores for original and structure-based alignments**. TM-score histograms obtained for original alignments in global (A) and local (B) evaluation modes without division into TP/FP. The data is shown for the 14516 most significant hits for each method (the number corresponds to COMA's hits up to *E*value = 0.01). Histograms in (C) and (D) show TM-score distributions for the same hits as in (A) and (B) respectively, but with TM-scores derived using DALI structural alignments.

It is a common knowledge that structure-based alignments are in general more accurate than those based on sequence (profile, HMM) comparison. Thus, we asked whether and, if so, to what extent alignments produced by individual methods in all-to-all comparison can be improved by using structure comparison? To answer this question, we realigned equal number of the top matching domain pairs for each method with DALI [26] and computed TM-scores for these alignments. Distribution of the original TM-scores and those based on DALI alignments are shown in Figure 5. One can see that the TM-score distribution derived using DALI structural alignments is strongly shifted towards higher TM-score values in both evaluation modes. In other words, DALI was able to improve significantly both the coverage and accuracy of alignments. Interestingly, after the realignment with DALI, the TM-score distribution is similar for all evaluated methods, except for PSI-BLAST, which remains significantly worse. These results indicate that the quality of homology detection for all of the evaluated methods (except for PSI-BLAST) is comparable. The observed differences in performance mostly come from differences in the alignment coverage and/or accuracy.

**Figure 6:**
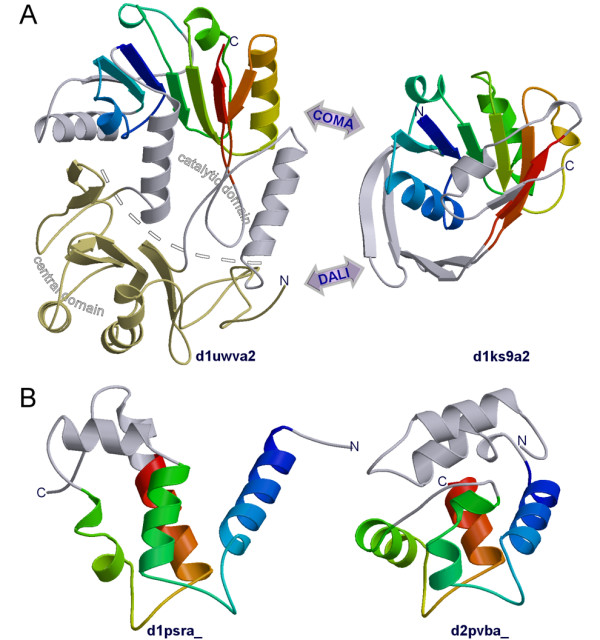
**Examples of COMA performing better than DALI, a structure-based method**. (A) COMA and DALI, each aligns d1ks9a2 with d1uwva2, but with different domains, catalytic and central, respectively. Coloring of d1ks9a2 and the catalytic domain of d1uwva2 corresponds to the progression of polypeptide chain from N-(blue) to C-terminus (red). (B) Homologous pair of EF-hand proteins, human psoriasin (d1psra_) and pike parvalbumin (d2pvba_).

Although structure-based methods such as DALI, often serving as providers of "gold standard" references, overall outperform profile (HMM)-based methods, they are not perfect either. Yet, in the reference-dependent evaluation, any disagreement with the "gold standard" is treated as an error. In contrast, using the reference-free setting, we can directly find cases when structure-based methods perform worse than the evaluated methods. Figure 6 provides a couple of such examples.

In the first example (Figure 6A), we consider two SCOP structures belonging to different folds that nevertheless have been identified by COMA as related at the domain level. It aligned ketopantoate reductase (d1ks9a2), a representative of Rossmann-fold (SCOP fold: c.2), and the methyltransferase (d1uwva2; c.66) RumA with statistically significant scores (*E*-value = 2.6e-3 and TM-score = 0.4262). DALI superimposed the corresponding structures producing Z-score of just 2, which is at the borderline of significance (structural matches with DALI *Z*-score lower than 2 are considered spurious [26]). Upon closer inspection, it turned out that COMA and DALI aligned the Rossmann-fold domain with different domains of RumA. COMA matched it with the catalytic domain representing a typical Rossmann-like SAM-dependent methyltranserase fold [33,34], reproducing previously established relationship. In contrast, DALI superimposed it with the iron-sulfur cluster-containing central domain, which has a different topology altogether [34]. Interestingly, when the isolated catalytic domain of RumA is compared to d1ks9a2, the resulting DALI alignment is assigned a much higher *Z*-score of 4.9. The corresponding TM-score = 0.4887 indicates that DALI has been able to improve over COMA's alignment, thus corroborating the relationship detected by COMA. However, note that this DALI result was obtained only due to a manual intervention. Were the original DALI results and the SCOP classification to serve as a reference, the distant relationship detected by COMA should have been considered either as false positive or at least dismissed as "unknown".

The second example (Figure 6B) involves human psoriasin (d1psra_) and pike parvalbumin (d2pvba_), a pair of clearly homologous proteins belonging to the same SCOP superfamily (EF-hand; a.39.1). While COMA correctly assigns a significant E-value (2.2e-4) to their alignment, DALI considers this to be a spurious match (Z-score = 1.9). Interestingly, both methods produce similar alignments, but the alignment by COMA (TM-score = 0.4725) is an improvement over DALI's (TM-score = 0.4234).

These two examples vividly illustrate the problems associated with the use of structure-based classification and/or alignments as "gold standard". With further improvement of homology detection methods the use of "gold standards" is going to be even more problematic.

## Discussion and Conclusions

In general, there are two major features that distinguish COMA from other sequence profile-profile comparison methods. The first one is position-dependent gap penalties. Although position-dependent gap penalties are commonly used in HMMs, to our knowledge, this study is the first one to implement them for comparison of sequence profiles. The second novel feature is the concept of the global score system. It enabled us to extend the analytical theory of statistical significance estimation developed for sequence comparisons into the comparisons of sequence profiles.

So far, a theory that could be as robust as that developed earlier for sequence comparisons [16] has been missing for profile-profile comparisons [35]. An earlier implementation of the analytical approach for sequence profile-profile comparisons [5] employs a number of simplifications in computing statistical parameters, thereby affecting their precision. One of the fundamental prerequisites for application of the Karlin-Altschul statistics [16] is the existence of the discrete score table. Here, to satisfy this requirement for profile comparisons we introduce a global score system that includes all the profile vectors from a profile database. Although the construction of the global score system has presented a serious challenge at the implementation level it has been successfully resolved. Thus, the introduced global score system provides a foundation for the accurate analytical estimation of statistical significance of ungapped profile alignments and for composition-based statistics. Gapped alignments, however, follow a different score distribution and, therefore, their statistical significance has to be estimated empirically. In the case of gapped alignments, we only focus on the tail of the score distribution, which is the most important region for significance estimation. In COMA, we fit it to EVD, but fitting to a slightly different distribution [23] is also possible.

Although in COMA the statistical significance of gapped alignments is estimated empirically, the resulting gapped alignments depend, albeit indirectly, on the Karlin-Altschul statistics. The dependence comes from the compositional adjustment of scores and, by definition, gap penalties, derived using the analytically solved statistical parameter values. Since the precision of these values strongly impacts the actual alignment of a profile pair, we believe that this significantly contributes to COMA's performance.

Along with algorithmic improvements, an important factor in the development of sequence profile-based distant homology detection methods is an effective evaluation framework. Historically, profile-based methods have been benchmarked against external "gold standard" protein classification schemes and reference structure-based alignments. However, profile-based methods have already advanced to a point, where very remote relationships can be detected. Often these distant relationships challenge the traditional hierarchical classification schemes such as SCOP and/or structure-based alignments (Figure 6). This is in line with the emerging view of "continuous" protein fold space and also with the observation that there is no single structure-based method that would always produce an optimal alignment (e.g. reviewed in [36]). In this study we used an evaluation method that does not depend on any reference classification or reference alignments and only requires the knowledge of protein 3D structures. In this evaluation scheme we ask a single question: how useful is the alignment between a query and a matching protein in reproducing the structure of the query. The closer are the protein structures and the more accurate is the alignment, the better score is assigned for the aligned pair.

Here, this general evaluation scheme has been applied in two different modes, global and local, each targeting different abilities of the evaluated methods. In a global evaluation mode an alignment is evaluated in respect to the entire protein domain. Therefore, in this mode not only more accurate but also more complete alignments are favored. Yet, a simple overprediction is not rewarded either, because if aligned regions are not structurally similar, their contribution to the total score is negligible. In contrast, the local evaluation mode is not concerned with the alignment coverage. It only measures how effective is the method in detecting and accurately aligning structurally similar protein segments, which can be very short.

It is obvious that there is an unavoidable trade-off in performance, evaluated using these two different modes. A method, optimized to produce very short accurate alignments that include only the structurally most similar segments (e.g. individual secondary structure elements) will fare well in the local evaluation mode, but would be very poor if evaluated at the domain level (global mode). In contrast, a method that tries to extend alignment into less structurally similar regions, may perform very well in the global mode, but will inevitably look worse if evaluated in the local mode. Certainly, for different tasks, different modes may be desired. For example, the accurate detection of short, structurally similar fragments is of great value in protein structure prediction by fragment assembly, regardless whether these fragments are homologous or simply reflect similar conformational preferences. However, if the method is to be used primarily for homology modeling or structural/functional annotation of protein domains, the good performance in the global mode is more appropriate.

COMA performs best at the protein domain rather than at the fragment level. This may be linked to position-dependent gap penalty scheme that allows the extension of alignment into structurally similar regions through sizeable variable regions (e.g. long loops). The shape of the ROC curve in the global evaluation mode shows that COMA is also good in distinguishing related from unrelated matches, an important property from the user's perspective. This property may be attributed to the new procedures of compositional adjustment of scores and statistical significance estimation. In conclusion, we hope that our new profile-profile comparison tool will be useful in studies of protein structure, function and evolution.

The COMA software package and a web server are freely available for academic use at http://www.ibt.lt/bioinformatics/coma/. The standalone package also includes programs for constructing the global score system and computing/scaling its statistical parameters for any profile database of interest.

## Authors' contributions

MM and ČV conceived of the study and designed research. MM carried out the research. MM and ČV analyzed data and wrote the manuscript. All authors read and approved the final manuscript.

## Supplementary Material

Additional file 1 **Supplementary table and figures**. Supplementary table provides the analysis of alignments defined as "true positives" by the TM-score criterion using two independent schemes ("SCOP" and "SCOP/DALI"). Supplementary figures provide supporting data for the results of methods evaluation. Figures S1 and S2 display ROC curves using correspondingly an alternative (log-scale) representation and alternative definitions for "true positives". Figure S3 provides a plot, showing how good is the agreement between the expected and observed P-values. Figure S4 represents evaluation results as Venn diagrams. Click here for file
