# Supplementary material for: Detection of distant evolutionary relationships between protein families using theory of sequence profile-profile comparison
Source: BMC Bioinformatics. 2010 Feb 17;11:89. doi: 10.1186/1471-2105-11-89 (PMC2837030; doi:10.1186/1471-2105-11-89)
Supplement: Additional file 1 — Supplementary table and figures. Supplementary table provides the analysis of alignments defined as "true positives" by the TM-score criterion using two independent schemes ("SCOP" and "SCOP/DALI"). Supplementary figures provide supporting data for the results of methods evaluation. Figures S1 and S2 display ROC curves using correspondingly an alternative (log-scale) representation and alternative definitions for "true positives". Figure S3 provides a plot, showing how good is the agreement between the expected and observed P-values. Figure S4 represents evaluation results as Venn diagrams. [file 1471-2105-11-89-S1.pdf]

Detection of distant evolutionary relationships between protein families using theory of sequence profile-profile comparison

Mindaugas Margelevičius, Česlovas Venclovas

Institute of Biotechnology, Graičiūno 8, LT-02241 Vilnius, Lithuania

Supplementary Table and Figures

Results

Table S1. Analysis of the TM-score criterion for homology detection

		TM-score	SCOP + DALI		SCOP	
		TPs	TPs	FPs	TPs	FPs
Global mode	**TM-score≥0.45**	13754	13748 (99.96%)	5 (0.04%)	11894 (86.48%)	953 (6.93%)
	**TM-score≥0.40**	17888	17864 (99.87%)	21 (0.12%)	14490 (81.00%)	1912 (10.69%)
	**TM-score≥0.35**	22928	22774 (99.33%)	137 (0.60%)	17168 (74.88%)	3504 (15.28%)
Local mode	**TM-score≥0.45**	33005	31803 (96.36%)	1174 (3.56%)	20401 (61.81%)	9100 (27.57%)
	**TM-score≥0.40**	39849	37575 (94.29%)	2232 (5.60%)	23092 (57.95%)	12439 (31.22%)
	**TM-score≥0.35**	46636	42727 (91.62%)	3856 (8.27%)	25233 (54.11%)	16419 (35.21%)

True positives (TPs) according to TM-score were obtained as follows: first, top 30 000 matches for every evaluated method (COMA, COMPASS, HHsearch, HHsearch w/o ss and PSI-BLAST) were pooled together and only non-redundant set was retained; next, pairs passing the indicated TM-score threshold were identified. Three sets of TPs as defined by different TM-score thresholds were then compared with the two independent classification criteria: “SCOP + DALI” and “SCOP”.

According to the “SCOP + DALI” criterion TP is defined to be a pair of proteins either from the same SCOP superfamily or the one producing a significant DALI *Z*-score (≥ 2). False Positive (FP) according to “SCOP + Dali” is considered to be a pair of proteins from different SCOP folds producing DALI *Z*-score < 2. A pair of proteins from the same SCOP fold but different superfamilies is ignored if its DALI *Z*-score < 2.

TPs according to the “SCOP” criterion are pairs from the same SCOP superfamily. FPs are pairs of proteins from different SCOP folds. Pairs of proteins coming from the same SCOP fold but different superfamilies are ignored.

ROC curves in log-scale

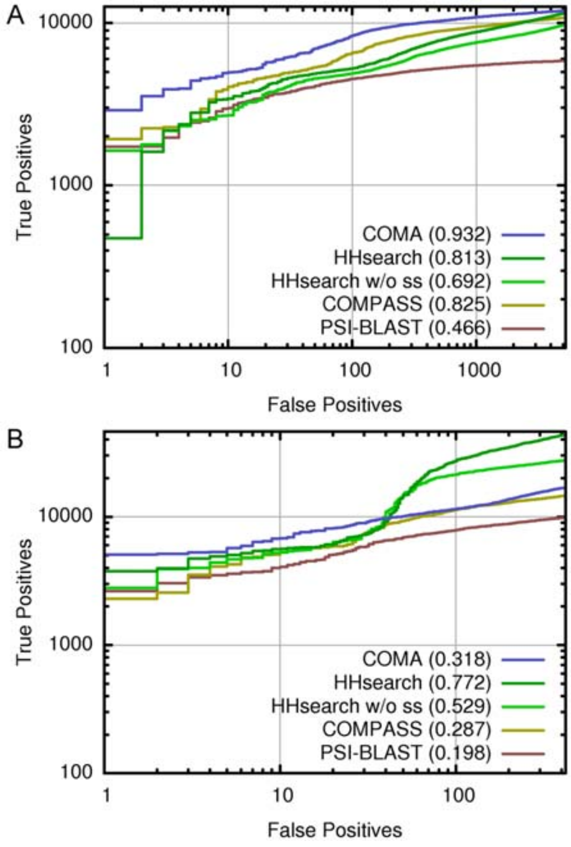

Figure S1. The ROC curves for each method in log-scale. (A) global and (B) local evaluation modes.

ROC curves based on alternative definitions for true positives

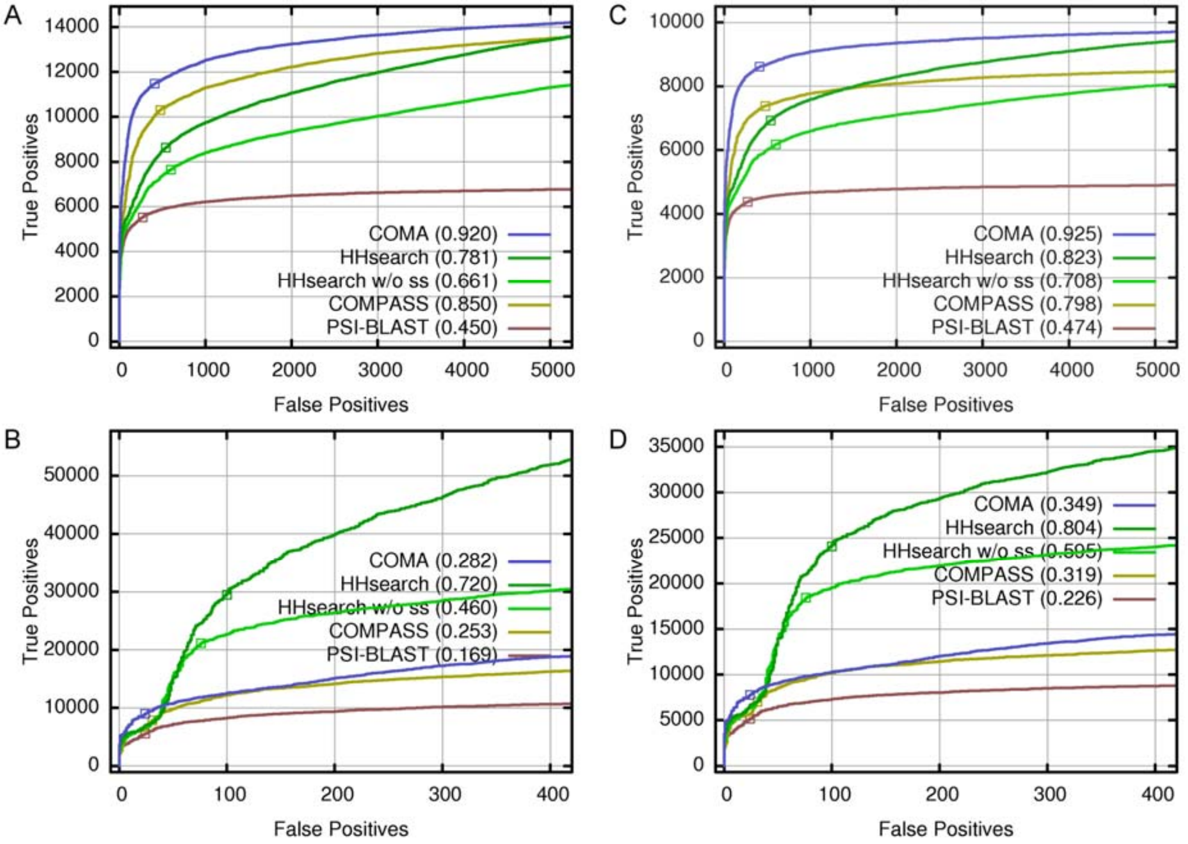

Figure S2. ROC curves for each method using alternative definitions for true positives. (A) global and (B) local evaluation modes using TM score ≥0.35 as the criterion for true positive; (C) global and (D) local modes with TM score ≥0.45 defining a true positive match. Empty squares in global mode ((A) and (C)) correspond to *E*-values of 0.01, 0.03, 0.0007 and 3e-6 for COMA, COMPASS, PSI-BLAST and HHsearch w/o ss, respectively; HHsearch probability is 97.6%. Empty squares in local mode ((B) and (D)) correspond to *E*-values of 8e-6, 1e-4, 1e-5, and 0.65 for COMA, COMPASS, PSI-BLAST and HHsearch w/o ss, respectively; HHsearch probability is 65.3%. These are the same *E*-values as in Fig. 3 in the main text.

Observed vs. Expected P-values

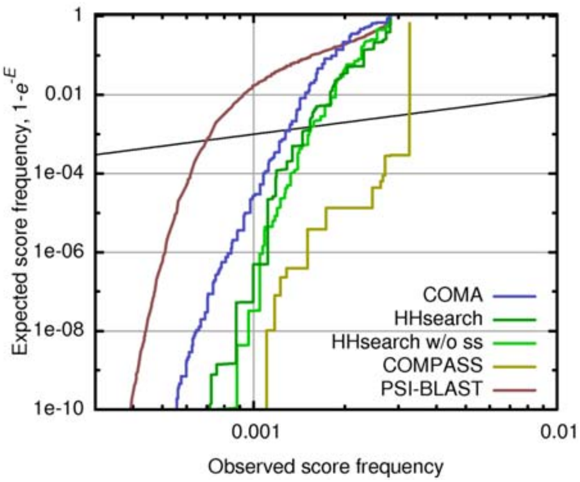

Figure S3. Plot of observed *P*-values vs. expected *P*-values expressed by the *E*-values of corresponding methods. Observed score frequency represents a relative number of alignments with the score equal or greater than the one corresponding to the specified *E*-value. For the HHsearch method (the version that uses secondary structure information) *E*-values are used instead of Probabilities. The ideal correspondence between the observed and the expected frequencies is indicated with a thin black line. For a particular method, the closer the slope and the intercept of the correlation curve is to the line of the ideal correspondence, the better statistical significance estimation is. In the area below the line of ideal correspondence, the methods underestimate *P*-values, while above the line they overestimate it. The plot reveals that all the methods tend to underestimate and overestimate *P*-values (they all use gaps in producing alignments), but to a different degree.

Venn diagrams

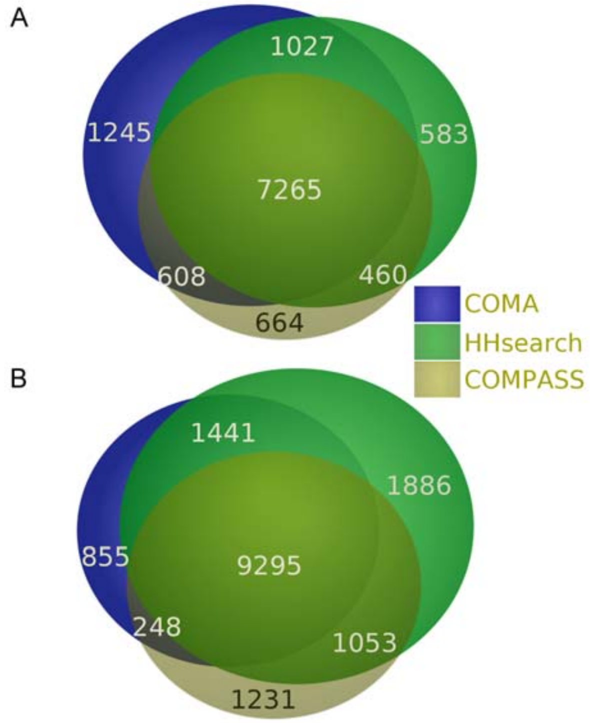

Figure S4. Venn diagrams of COMA, HHsearch and COMPASS evaluation results in (A) global and (B) local evaluation mode using TM-score≥0.4 as the criterion for true positive. The numbers of true positives are obtained from the 14516 most significant hits of each method, corresponding to COMA's *E*-value = 0.01.
